# Mammalian nuclear transplantation to Germinal Vesicle stage *Xenopus* oocytes – A method for quantitative transcriptional reprogramming

**DOI:** 10.1016/j.ymeth.2010.01.035

**Published:** 2010-05

**Authors:** R.P. Halley-Stott, V. Pasque, C. Astrand, K. Miyamoto, I. Simeoni, J. Jullien, J.B. Gurdon

**Affiliations:** Wellcome Trust/Cancer Research UK Gurdon Institute, The Henry Wellcome Building of Cancer and Developmental Biology, University of Cambridge, Tennis Court Road, Cambridge CB2 1QN, UK; Department of Zoology, University of Cambridge, UK

**Keywords:** GV, Germinal Vesicle, MBS, Barth-Hepes saline, PBS-BSA, phosphate buffered saline containing bovine serum albumin, SLO, streptolysin O, DTT, dithiothreitol, SuNaSp, sucrose, sodium chloride, spermine and spermidine, SuNaSp-BSA, SuNaSp with bovine serum albumin, RA, retinoic acid, ES, embryonic stem cell, ESRA, embryonic stem cell, treated with RA to differentiate and cease Oct4, Nanog and Sox2 transcription, *Xenopus laevis*, Nuclear transfer, Germinal Vesicle, Oocyte, Reprogramming, Microinjection, Quantitative PCR

## Abstract

Full-grown *Xenopus* oocytes in first meiotic prophase contain an immensely enlarged nucleus, the Germinal Vesicle (GV), that can be injected with several hundred somatic cell nuclei. When the nuclei of mammalian somatic cells or cultured cell lines are injected into a GV, a wide range of genes that are not transcribed in the donor cells, including pluripotency genes, start to be transcriptionally activated, and synthesize primary transcripts continuously for several days. Because of the large size and abundance of *Xenopus laevis* oocytes, this experimental system offers an opportunity to understand the mechanisms by which somatic cell nuclei can be reprogrammed to transcribe genes characteristic of oocytes and early embryos. The use of mammalian nuclei ensures that there is no background of endogenous maternal transcripts of the kind that are induced. The induced gene transcription takes place in the absence of cell division or DNA synthesis and does not require protein synthesis.

Here we summarize new as well as established results that characterize this experimental system. In particular, we describe optimal conditions for transplanting somatic nuclei to oocytes and for the efficient activation of transcription by transplanted nuclei. We make a quantitative determination of transcript numbers for pluripotency and housekeeping genes, comparing cultured somatic cell nuclei with those of embryonic stem cells. Surprisingly we find that the transcriptional activation of somatic nuclei differs substantially from one donor cell-type to another and in respect of different pluripotency genes. We also determine the efficiency of an injected mRNA translation into protein.

## 1 Introduction

An amphibian oocyte in meiotic prophase is a remarkable cell. The single full-grown *Xenopus* oocyte is 1.2 mm in diameter. Its tetraploid nucleus is 400 μm in diameter and contains hugely expanded lampbrush chromosomes [1], which are much more active in transcription than the nucleus of any other cell-type [2]. During its approximately nine months of growth, it accumulates many different proteins and RNAs needed for development of the embryo. Some of these components such as histones are in sufficient abundance in the mature oocyte to provide chromatin for the 12,000 nuclei of an embryo before transcription of the zygotic genome starts [3]. Each oocyte contains 10^12^ ribosomes, enough for the formation of a tadpole consisting of 10^5^ cells. Consequently, the anucleolate mutant, which has no ribosomal genes, survives till the feeding tadpole stage [4]. Therefore the *Xenopus* oocyte actively synthesizes huge reserves of components needed for development by prolonged and intense transcriptional activity.

Apart from its inherent interest, the oocyte is also extraordinarily useful as a cell to which macromolecules and complexes can be easily added. It is in effect a living test-tube, because a desired amount of any component can be introduced into its cytoplasm or its nucleus, and the injected oocyte can then be cultured in a salt solution for up to a month [5]. The value of *Xenopus* oocytes for injection first became evident when they were used to investigate the origin and identity of components required for DNA replication [6]. Since then, injected *Xenopus* oocytes have become increasingly useful for studies of protein regulation, RNA synthesis and the reprogramming of transplanted nuclei [7]. Here we review the characteristics of a *Xenopus* oocyte that enable it to switch the transcriptional program of transplanted somatic nuclei to that of an oocyte or embryo, a subject of much current interest in relation to the generation of embryo or stem cells from the differentiated cells of an adult.

Because one mature oocyte has the same volume as about 10^5^ somatic cells, conclusions can be drawn about the behaviour and properties of this single defined cell-type. It is hard, and perhaps impossible, to obtain such large numbers of identical somatic cells because most adult tissues consist of very many different cell-types, in different stages of the cell cycle, and in different states of differentiation or function.

## 2 Results

### 2.1 Preparation of oocytes and permeabilized cells

Oocytes should be removed from a frog, as whole ovary tissue, after terminal anaesthesia by submersion in 500 ml of water containing 1 g of Tricaine Methane Sulphonate (MS222) or by subcutaneous injection of 120 mg (in 300 μl) of MS222. This is the most humane method of termination available, with pithing being wholly inappropriate for use with *Xenopus*.

**Fig. 1:**
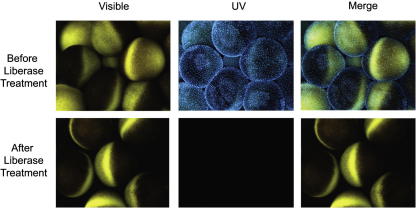
Chemical defolliculation of oocytes completely removes the oocyte follicular cells, illustrated here by Hoechst (UV) staining of follicular cell nuclei, before and after Liberase treatment.

When removed from a *Xenopus* ovary, each oocyte is surrounded by several thousand follicle and other cells (including blood vessels) and these make it very hard to penetrate the oocyte even with sharp injection needles. For many years, it was customary to peel off with forceps all except the innermost layer of follicle cells. Chemical defolliculation is much more convenient and faster, and in our experience is no more damaging to oocytes than mechanical defolliculation. This procedure is described by Astrand et al. [8]. Seven units of Liberase Blendzyme III or of Liberase TM Research Grade (Roche) (made up at 28 U/ml in H_2_O) are added to 3 ml of, well rinsed, loosely packed oocytes, which have been torn into groups of 30 or less, in 12.5 ml of MBS [9] (as a total volume) in a 50 ml Falcon tube. These are incubated with gentle rocking for 2–2.5 h at room temperature until the follicle cells have been separated (Fig. 1). The defolliculated oocytes are extensively washed in MBS and maintained in MBS, at 14–18 °C, for several days until required.

Donor cells must have their plasma membrane permeabilized if their nuclei are to be exposed to the GV or cytoplasm of a recipient oocyte. Compared to digitonin, lysolecithin, and some other reagents, Streptolysin O (SLO) (Sigma–Aldrich, S5265) can conveniently permeabilize the plasma membrane but not the nuclear membrane [10]. SLO was shown to be harmless when tested by traditional nuclear transfer to eggs [11]. Trypsinized cells must be washed thoroughly to remove as much serum as possible before SLO treatment, since proteins can inhibit SLO activity. SLO stock solutions are prepared in PBS (containing 0.01% BSA and 5 mM DTT) at 20,000 units/ml, warmed at 37 °C for 60 min, and stored in 25 μl aliquots at −80 °C. The exact procedure varies according to cell-type and preparation of SLO, but a standard procedure is to add 20 μl (400 units) of stored SLO to 10^6^ cells in 50 μl of SuNaSp [12], and incubate at 37 °C for 4 min. During or after this incubation, a few μl of cells can be added to 8 μl of 0.2% Trypan Blue in SuNaSp to determine the degree of permeabilization, seen as blue nuclei. We use preparations of cells that have been 95–99% permeabilized. As soon as permeabilization is sufficient, the reaction is stopped by adding an excess of SuNaSP BSA. A suspension of 10–25,000 permeabilized cells per μl in a suitable injection medium is used and this may be maintained on ice for a few hours until use.

### 2.2 Equipment for nuclear transfer to oocytes

The procedures and equipment for transplanting nuclei to oocytes have been described before [13,14]. Control of needle injection can be achieved by use of a Singer micromanipulator [9] and an Agla syringe [9], a Narishige (Narishige IM-300 microinjector, Narishige Scientific Instruments Lab., Japan), or Drummond (Drummond Nanoject, Drummond Scientific Company, USA) microinjector. We use a microscope stage equipped with temperature-controlled circulating water (usually at 15 °C) to keep the recipient oocytes cool during nuclear transplantation.

**Fig. 2:**
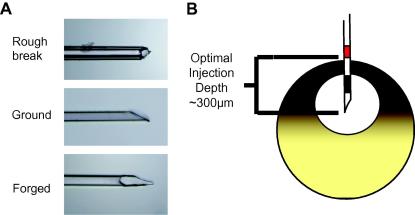
Typical needles used by members of our group (A). The ‘rough’ breaks are achieved by ‘nipping’ the needle tip with forceps. The needle can then be further sharpened by grinding or pulling on a micro forge, to give the ‘ground’ or ‘forged’ needle tips. GV targeting can be enhanced by marking the needles at 150–250 μm and 350–450 μm giving target depth marks on the needle shaft. (B) Diagram showing needle position of ‘minimum’ (black) and ‘maximum’ (red) depth marks on a needle in relation to a correctly targeted GV.

The needle profile is important for accurate GV targeting. A sharp glass needle (30–60 μm diameter tip) resembling that of a hypodermic needle is ideal. A number of different methods of achieving correctly shaped needle ends are available. The simplest of these involves crushing the tip until a sufficiently smooth but sharp edge is achieved. This type of needle can then be forged to give a sharper profile, or ground on a micro-grinder for a smooth and sharp finish (Fig. 2A).

The targeting of nuclei to the GV may be enhanced by marking the needle at roughly 250–300 μm from the tip with a fine laboratory marker to indicate the depth to which the needle should be inserted to deposit nuclei in the GV with a 80% or better success rate (Fig. 2B).

### 2.3 Injection into the Germinal Vesicle

**Fig. 3:**
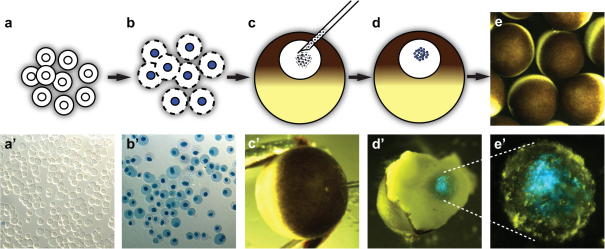
Nuclear transfer to *Xenopus* oocyte GVs. Cultured cells (a) are harvested, washed and permeabilised with SLO. Successful cytoplasmic membrane permeabilisation is detected by incorporation of Trypan Blue and cytoplasmic expansion (b, b′), whereas unpermeabilised cells do not incorporate the dye (a′). SLO permeabilised cells are directly transferred into the GV of stage V and VI *Xenopus* oocytes (c). Oocytes are held at a 45° angle using forceps, and nuclei are transferred directly by inserting the glass nuclear transfer pipette into the centre of the animal pole of the oocyte, until it is judged to have reached the inner part of the GV (c′). Oocytes are incubated in MBS medium, supplemented with 0.1% BSA, at 16–18 °C following transplantation (e). Successful targeting to the GV is revealed after oocyte dissection and Hoechst staining (in blue, d′). (e′) It shows an isolated GV containing injected nuclei stained with Hoechst (blue).

The major challenge in GV transplantations is to correctly target the nuclei to the interior of the GV and not the oocyte cytoplasm. This is done by positioning the oocyte with forceps, such that the pole of the animal hemisphere is at an approximately 45° angle to the vertical, facing the needle. The injection needle is then inserted directly into the animal pole to a depth of 250–300 μm and the desired volume of nuclear suspension dispensed into the GV (Fig. 3). An optimal injection volume is between 10 and 20 nl and this should be calibrated in the injection needle to deliver a constant number of nuclei to each oocyte.

Following nuclear transfer, oocytes are incubated at 16–18 °C (see below) in MBS, supplemented with 0.1% BSA, Gentamycin (1 μg/ml), Penicillin and Streptomycin (each at 10 μg/ml). The medium should be changed every day. Samples of four oocytes can then be removed when necessary, and frozen on dry ice in a microcentrifuge tube with as little medium as possible. They can then be stored at −80 °C for several weeks before processing.

### 2.4 Quantitative RT-PCR assays

**Fig. 4:**
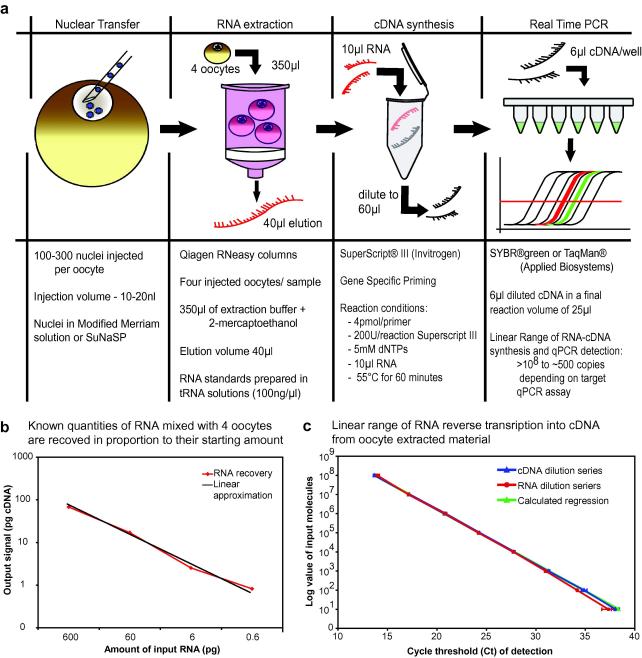
Schematic diagram of optimized RNA extraction, reverse transcription and qPCR, illustrating the conditions used for detecting transcripts following a nuclear transplant experiment (a). RNA is shown in red and cDNA in black. Critical parameters and output ranges are shown in the notes below the figures. Starting RNA quantities of a typical experimental series can be recovered from oocyte material in linearly proportional amounts following RNA extraction (b). Conversion of RNA to cDNA is linear across different input RNA amounts in an oocyte RNA background (c).

RNA extraction, reverse transcription (RT) and quantitative PCR (qPCR) on oocytes injected with nuclei have been extensively tested with the qPCR assays presented below to obtain a linear response to differing quantities of input RNA. The major findings of these experiments are summarized in Fig. 4.

Total RNA is extracted from samples of four injected oocytes using Qiagen RNeasy columns (74106, Qiagen), with a lysis buffer volume of 350 μl, containing 0.01% β-mercaptoethanol and on-column DNase I digestion for 15 min at 23 °C (Qiagen RNase free DNase I 79254). RNA is eluted in 40 μl of nuclease-free water. Reverse transcription is performed using SuperScript III (Invitrogen, 18080), with gene specific primers (0.2 μM final concentration each) and 10 μl of extracted RNA, as this has been determined to be the maximal volume of RNA that gives a linear PCR output. The resulting cDNA is diluted to 60 μl and 6 μl is used for qPCR. Real Time PCR is performed using the assay sets described in Table 1 either as SYBR®green assays or with modified oligonucleotide probes as TaqMan® assays (reagents from Applied Biosystems, primers and probes from Sigma–Aldrich and Applied Biosystems) on an ABI 7300 Real Time PCR Cycler using standard ABI cycling conditions (two-step PCR cycle: 94 °C for 15 s and 60 °C for 60 s). Reactions are performed in a total volume of 25 μl.

The oocyte is an unusual cell, containing a great abundance of stored RNA, protein and lipid. It is necessary to confirm that RNA extraction and cDNA synthesis of RNA give a linear signal output by qRT-PCR in response to differing quantities of target RNA in the oocyte background. Linear RNA recovery was determined by mixing a dilution series of an *in vitro* transcribed ‘spike’ RNA with four oocytes. RNA was extracted from these samples, reverse transcribed and the output quantities determined by qPCR. Fig. 4b shows the linear recovery of target transcripts from one such experiment. This is also in the quantity range that would be anticipated for rare transcripts from a reprogramming experiment. Similarly the linear conversion of purified RNA into cDNA was confirmed by reverse transcribing a dilution series of ‘spike’ RNA mixed with purified oocyte RNA. The result of this and a dilution series of the most concentrated sample were then analysed by qRT-PCR. Fig. 4c shows that the conversion of RNA to cDNA, under the conditions described above, is linear from 10^8^ to approximately 10^2^ input RNA molecules. Furthermore, this illustrates that a single RT reaction may be performed on one concentrated standard RNA sample and that the resulting cDNA may be diluted for use as a qPCR standard curve, as this gives almost identical PCR outputs when compared to the RNA dilution series.

Absolute quantitation is performed using cDNA made from standard reference RNA for the construction of standard curves, or from purified cDNA. The standard cDNA is synthesized from reference RNA in parallel with the samples of each experiment using 100 million molecules of standard RNA as a starting amount. RNA is always diluted in a tRNA (100 ng/μl) and RNase inhibitor cocktail (Roche, 03335399001) carrier solution. The resulting cDNA is then diluted in 10-fold steps to form the basis of the standard curve (again in carrier solution) for absolute quantification. The range of conversion from RNA to cDNA is linear from 10^8^ to approximately 10^2^ molecules using the protocol described above (data not shown). This allows a single concentrated RNA sample to be reverse transcribed and then diluted rather than each sample of the full dilution series needing to undergo RT. Reference RNA for each qPCR assay is produced by *in vitro* transcription of cloned 1–2 kb genomic fragments corresponding to a genomic region encompassing the qPCR amplicon. Primers containing the T7 and T3 transcriptional start sites used to clone these fragments are listed in Table 2. We should add that the appropriate selection of primers makes it possible to distinguish the production of primary unspliced transcripts from spliced transcripts of the same gene [17]. It has been shown that many transcripts from nuclei injected into *Xenopus* oocytes are not correctly spliced [18].

### 2.5 Nuclear suspension media

Permeabilized donor nuclei are suspended in a medium for injection that should ideally preserve donor nucleus integrity, prevent loss of donor protein and RNA and give a uniform suspension. Furthermore, it must be minimally damaging to the oocyte, particularly in the preservation of reprogramming activity, following injection into the oocyte GV.

We have tested a number of such preparations (Table 3) for this purpose. SuNaSp medium [12] is frequently used in this lab, giving good preservation of donor nuclear structure and clump-free suspensions. Callan’s medium is used for observing lampbrush chromosomes with minimal loss of these structures [19] and Merriam’s medium is suitable for collecting *Xenopus* GVs without significant loss of nuclear proteins [20–22]. Sox2 expression and oocyte survival were examined when mouse nuclei (embryonic stem [ES] cell or 4 day retinoic acid differentiated ES [ESRA]) were injected into the oocyte GV, after suspension in either SuNaSP, Callan’s medium or a modified Merriam’s medium. Permeabilized cells were suspended in 100 μl of each medium and then incubated for 3 h at room temperature or on ice. The prolonged incubation at room temperature prior to transplantation was intended to illustrate the impact of these media on reprogramming and oocyte survival, as we typically incubate nuclei at 4 °C. After incubation, cells were collected by centrifugation and suspended in a small volume (less than 10 μl) of each medium. Cell density was adjusted to be 300 cells per injection and 10 nl of each cell suspension was injected into oocyte GVs.

At all time points examined, Merriam’s medium supported higher expression of Sox2 and gave better oocyte viability (Table 4). This was true of both ES and differentiated ESRA cells. In contrast, SuNaSP gave lower oocyte viability and Sox2 activation. This negative effect seen with SuNaSP was largely negated when the cells were maintained at 4 °C, confirming that donor cells should be kept on ice before injection. In experiments where donor cells may need to be kept at room temperature, for example, in order to be treated with an enzyme or extract, Merriam’s medium may be the best choice.

### 2.6 The optimal number of nuclei to inject per oocyte

**Fig. 5:**
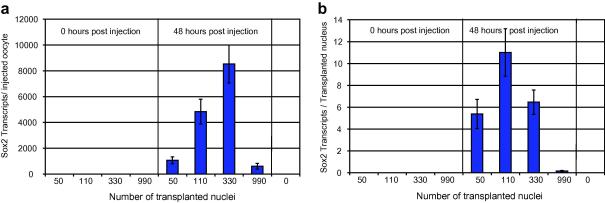
Reprogramming signal strength and efficiency are optimal between 100 and 300 nuclei. Transcription of Sox2 from different numbers of C2C12 nuclei injected into *Xenopus* oocytes and maintained at 16 °C for 48 h (a). Efficiency (absolute number of transcripts/transplanted nucleus) of Sox2 reactivation from different numbers of C2C12 nuclei injected into *Xenopus* oocytes and cultured at 16 °C for 48 h (b). *P* < 0.02, error bars are mean SD.

A single oocyte is capable of supporting the reprogramming and expression of mouse genes from many hundred injected mouse nuclei. To determine the optimal and acceptable numbers of injected nuclei that can be reprogrammed by the oocyte, we injected increasing numbers of nuclei, cultured at 16 °C, and analysed for Sox2 expression 48 h following injection (Fig. 5).

An injection quantity of around 300 C2C12 nuclei gives the optimal qRT-PCR signal. However, the maximal signal per nucleus, taken to be optimal for reprogramming, is seen at around 110 nuclei (Fig. 5b). As a result we recommend that a target injection number of 250 mammalian nuclei per oocyte be used for GV transplantation, as this gives a good balance between observing a strong qRT-PCR signal and optimal nuclear reprogramming.

### 2.7 Correction for the numbers of injected nuclei

**Fig. 6:**
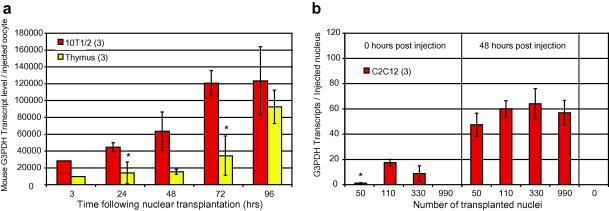
Accumulation of G3PDH transcripts over 4 days from oocytes injected with 10T1/2 or Thymus nuclei (a). Expression of G3PDH per injected C2C12 nucleus from different numbers of nuclei injected into *Xenopus* oocytes and maintained at 16 °C for 48 h (b). *P* < 0.02, except samples marked ∗*P* < 0.1, *n* = 3 (except first time point in A, where *n* = 1), error bars are mean SD.

The two major sources of technical variability between samples using qRT-PCR are related to RNA extraction efficiency and to the total amount of starting material in different samples. Both of these sources of error can normally be reduced by normalizing transcriptional results for a particular gene of interest to that of another gene that is assumed to be expressed at a constant level in all cell populations [23]. However, transcripts of all the candidate endogenous control genes tested to date for normalization of cell number are accumulated with time as illustrated for G3PDH in Fig. 6a. The increasing accumulation at different time points means that transcriptional results can not in this way be normalized to a time 0 value for G3PDH.

In contrast to normalization at different time points, it is possible to use a housekeeping transcript at one time point for standardization of nuclear injection numbers (Fig. 6b). As a caveat to this, however, the accumulation of transcripts is not exactly linear in proportion to cell number for G3PDH following transplantation into oocytes. G3PDH transcription is, nevertheless, relatively similar on a per nucleus basis between 100 and 300 nuclei (Fig. 6b) and can be used for normalization without introducing bias.

In the absence of an ideal cell number control, it is important to ensure that the desired number of nuclei is correctly injected. It is advisable for this reason to use a mechanised injector such as the Drummond or Narishige injectors described above and to ensure that nuclear suspensions are uniform (non-clumped). While the manual injector set up has a number of advantages over the mechanised systems it takes significant practice to learn to deliver consistently uniform injection volumes.

Further to this, we recommend that the number of nuclei being injected is always counted prior to starting an injection series. A convenient method to achieve this is to inject a few injection volumes of nuclei into MBS-BSA containing a small amount of DAPI and to count the number of cells for a representative injection under a fluorescent microscope. Furthermore, a PCR for a housekeeping gene should be included in any analysis to detect the presence of oocytes where the GV has been mis-targeted. A decrease by approximately 1.5 CTs is typically seen for G3PDH in our hands when one of the four oocytes has not been successfully injected.

### 2.8 Correction for variation in efficiency of RNA extraction

**Fig. 7:**
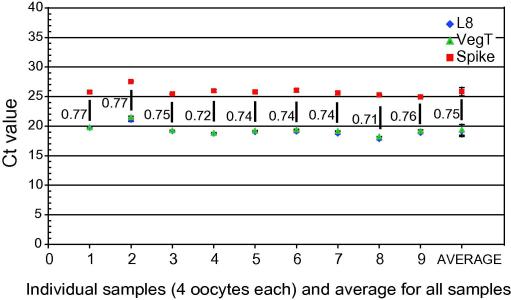
Real Time PCR Ct values for L8, VegT and an RNA “spike” gene for nine samples of four oocytes each. Numbers adjacent to bars indicate the ratio of the ‘spike’ Ct to mean L8 and VegT Ct values.

In contrast to the normalization of nuclear injection quantities, two oocyte-expressed genes (L8 and VegT) can be used to correct for variation in RNA extraction. This is illustrated in Fig. 7, where RNA was extracted from nine typical oocyte samples with added (“spiked”) RNA in the extraction buffer. The levels of VegT and L8 are in proportion to the added spike RNA in all samples, including two which consisted of small oocytes. This shows that these two genes are suitable for normalization in this context.

### 2.9 The rate of transcriptional activation differs according to donor cell-type

**Fig. 8:**
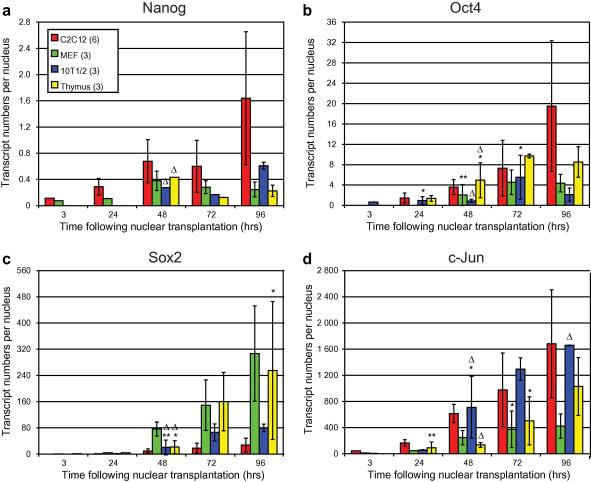
Transcription of Nanog (a), Oct4 (b), Sox2 (c) and c-Jun (d) initiates and increases over time following nuclear transplantation into the oocyte GV for each of the four donor nuclei types examined (see key). Samples were frozen at the indicated time points following transplantation in batches of 4 oocytes and examined for transcription of the genes by qRT-PCR. Error bars represent standard deviation of mean biological replicates (*n* is indicated in brackets for each cell-type in the key). *Δ* indicates one or more replicates discarded due to GV mis-targetting for that sample group. *P* < 0.5, unless marked ∗0.1 > *P* > 0.05 or ∗∗*P* > 0.1.

An interesting and unexpected finding is that different kinds of somatic cell nuclei differ substantially in the extent to which pluripotency genes are transcriptionally activated by oocytes. We have compared C2C12, mouse embryonic fibroblasts (MEFs), 10T1/2 and thymus nuclei in respect of Nanog, Oct4, Sox2 and c-jun transcription after transfer to oocytes (Fig. 8a–d). It is evident that transcription increases progressively with time for c-jun. These transcripts generated per nucleus are highest for C2C12 and 10T1/2 nuclei (about 1600 transcripts/nucleus after 3 days). Other work from our laboratory has shown us that c-jun transcripts have a short half-life of a few hours; these increasing levels of c-jun transcripts seem therefore to represent *de novo* transcription after transfer to an oocyte, as donor cell levels of c-Jun become undetectable following SLO permeabilization unlike many more stable transcripts. C2C12 nuclei generate a high level of Nanog and Oct4 transcripts and a low level of Sox2 transcripts compared to other nuclei. In contrast, mouse embryo fibroblasts and thymus nuclei generate a high level of Sox2 transcripts compared to 10T1/2 and C2C12. Comparing the ratio of transcripts of Nanog to those of Sox2 at 96 h, there is an over 50-fold difference between C2C12 versus MEF and thymus nuclei (Fig. 8a and c). In all our experiments, we find that the absolute level of transcript production from transplanted nuclei (assuming similar half-lives) is very low for Nanog, but much higher for Oct4, Sox2 and c-jun in that order.

**Fig. 9:**
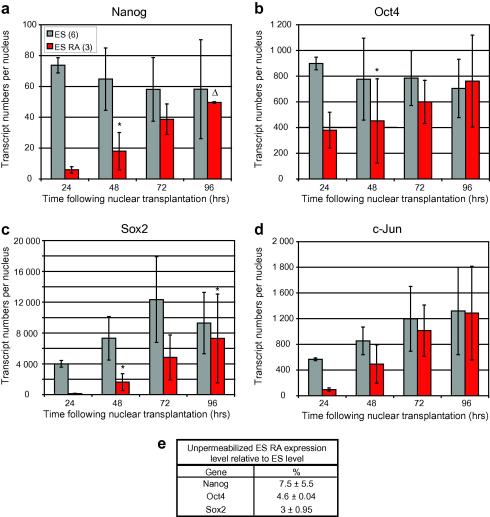
Expression of Nanog (a), Oct4 (b), Sox2 (c) and c-Jun (d) from oocytes injected with either ES or retinoic acid differentiated ES (ESRA) nuclei (see key). (e) Transcript values for Nanog, Oct4 and Sox2 in wholes ESRA cells as compared to whole ES cells prior to permeabilization. This illustrates the typical differences between ES and 4 day retinoic acid-treated ESRA cells. Error bars represent standard deviation of mean biological replicates (*n* is indicated in brackets for each cell-type in the key). *Δ* indicates one or more replicate discarded due to GV mis-targetting for that sample group. *P* < 0.5, unless marked ∗0.1 > *P* > 0.05 or ∗∗*P* > 0.1.

In Fig. 9, we make similar transcript comparisons for ES and ESRA nuclei in oocytes. We point out that transplanted ES nuclei (in fact permeabilized cells) have a high content of Nanog and Oct4 transcripts. Sox2 and c-jun transcripts are almost absent from ES and ESRA nuclei at the time of transfer to oocytes (not shown), owing to loss of these unstable transcripts following permeabilization; a comparison at this time point is meaningless. Fig. 9e shows the typical Nanog, Oct4 and Sox2 transcript levels seen in 4-day RA-differentiated ES cells prior to permeabilization. This illustrates the reduced level of these three transcripts in RA-ES cells in comparison to growing ES cells. For both ES and ESRA nuclei there is a steady accumulation of all these transcripts after transfer to oocytes during four days. As with transplanted somatic nuclei (Fig. 8), we see a progressive increase in transcript abundance in the sequence Nanog, Oct4, Sox2 and c-jun.

When we compare the extent of transcriptional activation in somatic versus ES or ESRA nuclei, it is remarkable how much more strongly oocytes activate the three pluripotency genes in ESRA nuclei than in somatic nuclei. This difference is up to 50–100 times (Table 5). On the other hand, the housekeeping gene c-jun is transcribed to about the same extent in transplanted ESRA and somatic nuclei (Table 5). This huge difference illustrates strikingly the difference between an active housekeeping gene and the developmentally repressed pluripotency genes in somatic cells. An oocyte can reverse the repressed state to a large extent, but nevertheless the process of cell differentiation entails a substantial resistance to the reprogramming activity of an oocyte.

### 2.10 The translational efficiency of mRNAs injected into oocytes

It is often desirable to overexpress a gene or its downstream target in oocytes as a test of function. For maternally expressed genes in oocytes there is often a very large protein content. It can therefore be useful to know how much mRNA should be injected to generate as much or more new protein compared to the maternal amount already present in the oocyte and its GV. Using mRNA encoding Lac-GFP we found that an injection of 3 × 10^9^ mRNA molecules to the cytoplasm of an oocyte yielded 2.2 × 10^10^ molecules of protein in the GV in 16 h at 16 °C. This rather large protein (55 kDa) enters the GV slowly and the amount in the GV in this case was only 50% of the total in the cell. This production rate, assuming that the protein is wholly stable over this time, amounts to a synthesis rate of about 200 molecules of protein per day per injected mRNA molecule (at 16 °C). This is reasonably in accord with a previous estimate of at least 170 molecules of protein per day per injected mRNA molecule for globin mRNA translation in oocytes using an entirely different procedure [24].

## 3 Discussion

The large size of an amphibian oocyte makes it exceptionally well suited for the introduction, by microinjection, of any macromolecule, multicomponent assembly, or organelle into either the cytoplasm or nucleus (Germinal Vesicle), in a harmless way, and the injected oocyte can be cultured in a defined medium for days or weeks. In this way, it resembles a living test-tube. In this article, we have summarized past information and contributed new results all of which concern the use of *Xenopus* oocytes for analysing the transcriptional reprogramming of transplanted somatic nuclei. We have emphasized the value of quantitative transcriptional assays to demonstrate remarkable differences in the reprogramming of unrelated somatic cell nuclei. These quantitative assays will further help to better define the molecular mechanisms underlying these differences.

Future developments in this area can be envisaged in two important directions. One is real time imaging. The oocyte nucleus (GV) can be manually isolated, and maintained in a non-aqueous medium [22]. Since our isolated GV is translucent, events associated with the reprogramming of injected nuclei can be seen in real time, often in combination with fluorescence. FRAP analysis of protein mobility is a good example of this direction of work [25].

Another future direction is to take advantage of the large amount of oocyte material available. One frog has about 25,000 oocytes, and each *Xenopus* oocyte is 4000 times larger than a mammalian oocyte or egg. Egg extracts have been very successful in *Xenopus* for analysing DNA replication [26]. At present, no *Xenopus* oocyte extract has been able to satisfactorily reproduce transcriptional reprogramming entirely *in vitro*. However, in combination with the re-sealing of extract treated permeabilized cells [27–29] new gene transcripts can be seen. It may be possible, in time, to find ways of obtaining new transcription entirely with *in vitro* extracts, using the *in vitro* results presented here to guide the making of functional extracts. This would greatly help attempts to identify the reprogramming molecules of oocytes and eggs.

## Figures and Tables

**Table 1:** Real Time PCR Primers for the detection of mouse transcripts in a Xenopus laevis transcript background.

Assay name	Target gene	Target sequence (accession)^a^	Primer name	Sequence (5’ to 3’)^b^	Supplier	Concentration (mM)^c^^,^^d^	Amplicon length (bp)	Linear Range (RNA)^e^	Detection Limit (DNA)^f^	Origin
q11Nanog	*M. musculus* Nanog	NC_000072	q11NanF	TCTCTCAGGCCCAGCTGTGT	Sigma–Aldrich	900	65	10^8^–500	ND	Gurdon Lab
			q11NanR	GCTGGAGGCTGAGGTACTTCTG	Sigma–Aldrich	900				
			q11NanFAM	{6FAM}-CACTCAAGGACAGGTTT-{NFQ-MBP}	Applied Biosystems	250				

qOct4	*M. musculus* Pou5f1	NC_000083	qOct4F	GAAGGGCAAAAGATCAAGTATTGAG	Sigma–Aldrich	650	78	10^8^–500	10	Gurdon Lab
			qOct4R	GCCCCCCCTGGGAAAG	Sigma–Aldrich	1300				
			qOct4PFAM	{6FAM}-CCCAACGAGAAGAGTATGAGGCTACAGGGAC-{BHQ-1}	Sigma–Aldrich	250				

q3Sox2	*M. musculus* Sox2	NC_000069	q3Sox2F	TCAGGCTGCCGAGAATCC	Sigma–Aldrich	700	97	10^8^–250	30	Gurdon Lab
			q3Sox2R	TCAAACTGTGCATAATGGAGTAAAAAC	Sigma–Aldrich	900				
			q3Sox2pFM	{6FAM}-TGAACTAATACCATCCTTATAAC-{NFQ-MBP}	Applied Biosystems	250				

qJun	*M. musculus* c. Jun	NC_000070	qJunF	CCTGTCCCCTATCGACATGG	Sigma–Aldrich	900	93	10^8^–500	ND	RTDB⁎
			qJunR	CTTTTCCGGCACTTGGAGG	Sigma–Aldrich	700				
			qJunFAM	{6FAM}-TCCTCATGCGCTTCCTCTCTGCCT-{BHQ-1}	Sigma–Aldrich	250				

q5G3PDH	*M. musculus* G3PDH	AC_000028	q5G3PDHF	CATGGCCTTCCGTGTTCCT	Sigma–Aldrich	100	55	10^8^–1000	ND	Surani⁎⁎ Lab
			q5G3PDHR	GCGGCACGTCAGATCCA	Sigma–Aldrich	100				
			q5G3FAM	{6FAM}-CCCCAATGTGTCCGTC-{NFQ-MBP}	Applied Biosystems	250				

q3VegT	*X. laevis* VegT	NM_001088202	q3VegTF	CATCGCTACAAGCCCAGGTT	Sigma–Aldrich	80	95	10^8^–100	10	Gurdon Lab
			q3VegTR	TCTGTCTCTGGGAAGCTAAACACTT	Sigma–Aldrich	80				
			q3VegTpVM	{VIC}-ATGACATGTACAATTCTCCA-{NFQ-MBP}	Applied Biosystems	250				

qL8	*X. laevis* L8	NM_001086996	qL8F	AGGTCGTGCCTACCATAAATACAAG	Sigma–Aldrich	100	93	10^8^–500⁎⁎⁎	100	Gurdon Lab
			qL8R	ACCACCGAAGGGATGTTCAAC	Sigma–Aldrich	100				
			qL8pVM	{VIC}-TGGTGTGGCTATGAAT-{NFQ-MBP}	Applied Biosystems	250				

a Entrez gene accession numbers.

b Characters in parentheses represent 5′- or 3′-oligo modifications: BHQ-1 and NFQ are non-flourescent quenchers, MBP is a Minor-groove Binding Protein.

c Oligo primer concentrations for Taqman assays – oligo concentrations for the first four assays are optimized for maximal detection. Latter 3 are concentration limited to give minimal total amplification without compromising Ct values, to allow for multiplexing if desired.

d SYBR Green experiments performed with oligos at a concentration of 100 nm. All assays give a single sharp dissociation curve peak when template is present and no amplification in oocyte background when no target template is present.

e Linear range of detection (numbers of molecules), determined using purified *in vitro* transcribed target RNA as a source of starting material. For example 108–500 means linear detection from 108 molecules to 500 molecules.

f Limit of detection determined using purified target cDNA as starting material for assay. ND is not determined.

⁎ RTPrimerDB [16].

⁎⁎ Primer sequences from Ref. [15], probe from Gurdon Lab.

⁎⁎⁎ Determined with DNA starting material and not RNA.

**Table 2:** Primers for cloning genomic fragments for *in vitro* transcription of RNA used as standard for qRT-PCR.

Fragment	Direction	Sequence (5′–3′)^a^	Amplicon (kb)
Nanog	Forward	CTCC**TAATACGACTCACTATAGGG**TGCCTCTTCAAGGCAGCCCTGATTCT	2
	Reverse	GTAG**AATTAACCCTCACTAAAGGG**TTGTGGGGTGCTAAAATGCGCATGGC	
Oct4	Forward	AGGA**TAATACGACTCACTATAGGG**AGCTAGAACAGTTTGCCAAGCTGCTG	1.4
	Reverse	CTGGTG**AATTAACCCTCACTAAAGGG**AGAGCCCAGAGCAGTGACGGGAAC	
Sox2	Forward	GCCCGC**TAATACGACTCACTATAGGG**ATGATGGAGACGGAGCTGAAGCC	2.1
	Reverse	TGTC**AATTAACCCTCACTAAAGGG**ATCTCAAACTGTGCATAATGGAGTAA	
G3PDH	Forward	CAGCTC**TAATACGACTCACTATAGGG**ATTTGGCCGTATTGGGCGCCTGG	1.4
	Reverse	CTTG**AATTAACCCTCACTAAAGGG**TTTCTTACTCCTTGGAGGCCATGTAG	
c-Jun	Forward	CGC**TAATACGACTCACTATAGGG**ATGACTGCAAAGATGGAAACGACCTTC	1.1
	Reverse	TTGC**AATTAACCCTCACTAAAGGG**TTCTCAAAACGTTTGCAACTGCTGCG	
VegT	Forward	GTGATA**TAATACGACTCACTATAGGG**AATGAGAAACTGCTGTCGGGAATG	1.4
	Reverse	GTGGAT**AATTAACCCTCACTAAAGGG**TTACCAACAGCTGTAGGGGAAGAG	

a T7 and T3 RNA promoter sequences are shown in bold. T7 is used on the forward primer for synthesis of *in vitro* RNA that mimics a portion of the corresponding mouse primary transcript.

**Table 3:** Composition of Nuclear Suspension Media.

Reagent	Nuclei suspension medium		
	SuNaSP [12]	Modified Merriam’s medium [20]	Callan’s medium (B) [19]
Sucrose	250 mM		
KCl		75 mM	29.2 mM
MgCl_2_		2 mM	
KH_2_PO_4_			0.3 mM
NaCl	75 mM		5.8 mM
HEPES–KOH (pH 7.5)		20 mM	
Polyvinylpyrrolidone		2%	
BSA^a^	(3%)	(1%)	
SpermIdine	0.5 mM		
Spermine	0.15 mM		
2-Mercaptoethanol		2 mM	

a BSA included for final injection suspension but not for permeabilization.

**Table 4:** Effect of different media for donor cell suspension on oocyte viability and Sox2 reactivation.

Type of donor cell^a^	Type of medium	Incubation temperature^b^	Survival (%) (66 h at 16 °C)	Fold increase of Sox2 transcript^c^^,^^d^
RA-ES	SuNaSp	23 °C	16	n.d.
RA-ES	SuNaSp	4 °C	32	1
RA-ES	Callan’s medium	23 °C	88	0.99
RA-ES	Merriam’s medium	23 °C	80	6.42
ES	SuNaSp	23 °C	16	n.d.
ES	SuNaSp	4 °C	28	2.53
ES	Callan’s medium	23 °C	68	4.83
ES	Merriam’s medium	23 °C	84	18.38

a RA-ES represents retinoic acid-treated (4 days) embryonic stem (ES) cells.

b Permeabilized nuclei were incubated at either 4 °C or 23 °C for 3 h prior to injection.

c n.d. represents ‘not determined’.

d Sox2 transcripts were measured by qPCR using samples of 4 oocytes, each injected with ES or RA-ES nuclei and incubated for 66 h prior to analysis. Expression is relative to SuNaSp at 4 °C.

**Table 5:** Maximum level of gene reactivation in different cell-types relative to the equivalent value seen in samples injected with ES nuclei (%).

	Relative transcript expression^a^			
Donor cell-type^b^	c-Jun	Sox2	Nanog	Oct4
C2C12	516.3	0.3	2.8	2.8
MEF	130.0	3.3	0.6	0.6
Thymus	125.6	0.9	1.0	0.7
10T1/2	78.0	3.0	0.7	1.2
ESRA	230.5	78.5	85.2	108.1

Ratio between mean somatic and ESRA level	1.1	42.1	66.6	81.1

a Values are shown as % of that for injected ES cell nuclei.

b Oocytes injected with 250 nuclei and cultured for up to 4 days at 16 °C.
